# Fat-Free Mass Is Positively Associated With Urine Specific Gravity in Athletes and Active Adults: A Quantitative Review

**DOI:** 10.1155/tsm2/8827027

**Published:** 2024-12-09

**Authors:** Patrick B. Wilson, Ian P. Winter

**Affiliations:** Human Performance Laboratory, School of Exercise Science, Old Dominion University, Norfolk, Virginia, USA

**Keywords:** body composition, creatinine, skeletal muscle, sport, urea, uric acid

## Abstract

**Background:** Urine specific gravity (USG) is a commonly used assessment method to estimate the prevalence of hypohydration, typically based on a common threshold of ≥ 1.020. Some research has shown that USG can vary based on body size and composition, but the evidence to date is limited. This review examined whether an association between fat-free mass (FFM), a component of body composition, and USG could be detected among published articles that have reported these variables in athletes and physically active individuals.

**Methods:** By searching Google Scholar, the authors identified a large number of published articles (*n* = 161) reporting anthropometrics and USG. Only articles reporting data on adult samples of athletes or physically active individuals were included. Given differences in body composition and FFM between men and women, articles that did not report data separately for each sex were excluded. Spearman's rank correlation coefficient (*ρ*) was used to assess the association between variables.

**Results:** In men, FFM showed a significant, weak-sized positive association with USG (*ρ* = 0.36, *p* < 0.001). Among women, FFM showed a significant, moderate-sized positive association with USG (*ρ* = 0.57, *p*=0.006). When results were combined independent of sex, the association between FFM and USG remained significant (*ρ* = 0.38, *p*  < 0.001).

**Conclusion:** Athletes with larger amounts of FFM are more likely to have elevated USG. Protein and muscle metabolites such as creatinine, urea, and uric acid likely play some role in the observed relationships. If USG continues to be utilized in sport settings, more research is needed to determine if development of FFM-specific USG thresholds may be more appropriate for determining hydration status.

## 1. Introduction

Maintaining adequate hydration has long been regarded as important for optimizing an athlete's training quality and performance, particularly in thermally stressful environments [1]. Accurately assessing whether an athlete is adequately hydrated, however, remains as an ongoing challenge. Indeed, there is no universally accepted gold standard measure of hydration, and many practical, field-based methods have poor or uncertain validity [2]. Despite these issues, practitioners often rely on convenient methods such as urine specific gravity (USG) and urine color to assess the hydration status of their athletes. In particular, the use of USG is commonplace because the cost of portable urine refractometers is relatively low compared to other tools, and urine is a more accessible body fluid than blood. In addition, USG provides a more objective and reliable assessment than urine color [3].

Beyond its use in practice, USG is also frequently utilized by researchers to estimate the prevalence of hypohydration in athletic populations. The most common threshold used to define hypohydration is a USG ≥ 1.020 [2]. When this threshold is applied, studies of athletes tend to report high estimates of hypohydration before the onset of training and competition. For example, according to a systematic review, approximately one-half and two-thirds of female and male soccer players, respectively, are supposedly hypohydrated before exercising [4]. Likewise, slightly over half of 29 National Basketball Association players started summer league games in a supposedly hypohydrated state [5]. An even higher prevalence of alleged hypohydration, 75%, was found among division III NCAA male athletes in one study [6], and another found that nearly 80% of division II male and female basketball players had a USG > 1.020 prior to practice [7].

The abovementioned body of research seemingly suggests that hypohydration is very prevalent among athletic populations, even prior to beginning exercise. Several studies, however, have raised a potential concern with using this 1.020 threshold across individuals of different body sizes [8–10]. More specifically, having larger absolute amounts of fat-free mass (FFM) may be associated with higher USG levels, and this association may partly arise from the fact that muscle is a source of the creatine breakdown product creatinine, which is excreted in the urine [11]. The implication of this finding is that athletes who have larger amounts of muscle could be at greater risk of being classified as hypohydrated when they are not in fact hypohydrated. However, additional research is needed to confirm this suggestion.

To date, research that has directly evaluated the relationships between USG, body size, and body composition (particularly FFM) in athletes is limited [9]. Furthermore, existing studies have not sampled a broad range of athletes in terms of type of sport and body size. In order to document any associations between FFM and USG, it would be advantageous to look at athletes across the full spectrum of body size. Therefore, the purpose of this review was to examine whether an association between FFM and USG could be detected among a large selection of published studies that have reported these variables in athletes and physically active individuals. It was hypothesized that there would be a positive association between absolute amount of FFM and USG.

## 2. Materials and Methods

Although systematic reviews are commonly used to identify investigations on a scientific topic, the present review did not intend to locate every single study that has reported USG along with body size and body composition data. Rather, the goal was to search for and identify a large selection of studies (> 100) in order to examine if significant associations could be detected between the reported variables, principally FFM and USG.

The primary means of searching for relevant articles was Google Scholar. Articles that reported USG along with relevant anthropometrics of interest (body mass, body mass index (BMI), FFM, fat mass, and body fat percentage) were considered for inclusion. Only articles that reported data on athletes or physically active individuals were included. In addition, only articles that primarily reported data on adults (≥ 18 years old) were included; this was determined based on the reported average age of participants, any listed aged-related inclusion criteria, or other relevant terms describing the likely age of the sample (e.g., university athletes). Given the known differences in body composition and FFM between men and women, articles that did not report data separately for each sex were excluded. There were no restrictions on study design (observational vs. experimental), although included studies had to present original data. Peer-reviewed articles in English with a full text available were eligible for inclusion.

A wide range of search terms (body composition, lean mass, lean body mass, body fat, urine specific gravity, specific gravity of urine, and USG) related to the variables of interest and populations of interest (athletes, exercisers, runners, cyclists, triathletes, soccer, basketball, football, etc.) was used to find relevant articles. The search process continued until a large number of articles was identified (*n* = 161) and it became difficult to find any new applicable articles.

As relevant studies were identified, pertinent information was extracted and entered into a spreadsheet (Supporting Information 1) by the first author (PW). Extracted information included the type of athlete/exerciser, sample size, and sex distribution, as well as mean values for age, height, body mass, BMI, body fat percentage, FFM, body fat mass, and USG. In some cases, one variable was calculated from others (e.g., body mass calculated from BMI and height, BMI calculated from mass and height, and FFM calculated from total mass and percentage body fat). When an individual article reported statistics from multiple athlete types/subsets/groups, data for each type/subset/group were summarized separately. In addition, some studies reported multiple USG values (e.g., at different timepoints or using different methods such as spot or 24-h collections); in these cases, the specific USG value that was used for this analysis has been specified in the spreadsheet (Supporting Information 1). In general, only resting or pre-exercise USG values were used in the analysis, with values taken post-exercise excluded from consideration. Other contextual notes about the data as well as the methods/instruments that were used to quantify USG and body composition are also included in Supporting Information 1

After all the pertinent information was entered into the spreadsheet, it was then double-checked for errors by a second author (IW). Next, one of the authors (PW) checked the entries for possible cases where data were duplicated between two or more articles. This was done by examining author names and details of the sample (location of the study, year(s) of data collection, details of the participants, etc.). In addition, several variable columns in the spreadsheet were searched for duplicate numerical values; studies that reported exactly the same values out to 0.1 or 0.01 were evaluated as possible duplicates.

The distribution of continuous-type variables (age, height, body mass, BMI, percent body fat, fat mass, FFM, and USG) was examined with histograms and Q–Q plots. All the variables except USG exhibited a right skew, which was not made normal through data transformations. Thus, descriptive data are reported as median (25^th^–75^th^ percentile). In order to evaluate whether any variables of interest (age, height, body mass, BMI, percent body fat, fat mass, and FFM) were associated with USG, the Spearman's rank correlation coefficient (*ρ*) was utilized given the right-skewed nature of the data. Effect sizes for *ρ* of 0.0–0.19, 0.20–0.39, 0.40–0.59, 0.60–0.79, and 0.8–1.0 were deemed very weak, weak, moderate, strong, and very strong, respectively. The sample sizes varied for each correlation test since not all studies reported all the variables of interest. The analyses were initially carried out for each sex separately, followed by a combined sex analysis to see if the effects persisted. A two-sided *p* value of ≤ 0.05 was considered as the threshold for statistical significance.

## 3. Results

Details of the identified reports and extracted data can be found in Supporting Information 1. Descriptive data for the variables of interest are reported in Table 1. Overall, there were more estimates available for men, with USG having the largest number (*n* = 171) and fat mass and FFM having the lowest number (*n* = 91). Among women, the variable with the most estimates was USG (*n* = 59), while the variables with the fewest available estimates were body fat percentage, fat mass, and FFM (*n* = 22).

Table 2 shows the Spearman *ρ* correlations between USG and potential predictor variables. In men, body mass, BMI, and FFM all showed significant (*p* < 0.05) positive (weak-sized) associations with USG. Age, on the other hand, showed a negative (weak-sized) association with USG. The effect sizes were largest for age (*ρ* = −0.34) and FFM (*ρ* = 0.36).

Among women, age and FFM were the only variables that showed significant (*p* < 0.05) associations with USG. Age showed a moderate-sized negative correlation (*ρ* = −0.57) with USG while FFM also showed a moderate-sized positive correlation (*ρ* = 0.57) with USG.

**Figure 1:**
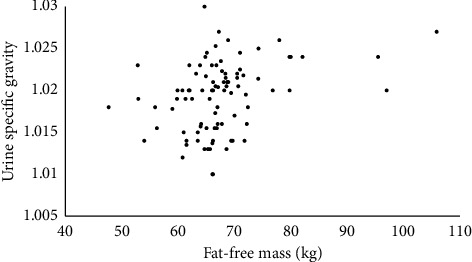
Associations between urine specific gravity (USG) and fat-free mass (FFM) among men.

**Figure 2:**
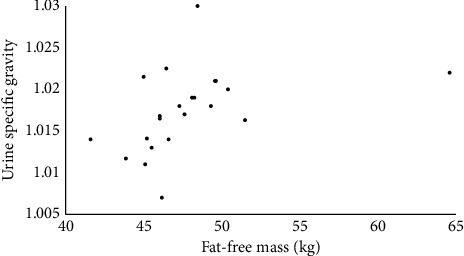
Associations between urine specific gravity (USG) and fat-free mass (FFM) among women.

**Figure 3:**
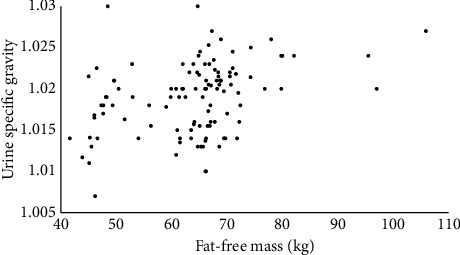
Combined sex associations between urine specific gravity (USG) and fat-free mass (FFM).

The association between FFM and USG is represented visually, for each sex separately, in Figures 1 and 2. Based on the notion that the absolute amount of FFM should associate with USG independently of sex, an additional correlation was run with data for men and women combined (*n* = 113). The Spearman *ρ* remained significant (*ρ* = 0.38, *p* < 0.001), and the relationship is shown visually in Figure 3.

## 4. Discussion

This study's aim was to examine whether an association between anthropometric variables and USG could be detected among many (> 100) published studies that reported those variables in athletes and physically active individuals. Using a large, broad sample of athletes of different sports and body sizes, this quantitative review found that FFM and USG have a weak-to-moderate-sized positive correlation. While BMI and body mass were also positively associated with USG (at least in men), the lack of significant association between fat mass and USG means that FFM is likely the main driver of these associations. In addition, neither body mass nor BMI were associated with USG among women, while FFM did show a moderate-sized positive association with USG. In total, the results support the notion that, among the anthropometric variables studied, FFM is the most important determinant of USG. However, differences between these associations were apparent between men (weak) and women (moderate), as depicted by Figures 1 and 2, respectively. This suggests that the FFM–USG connection may depend on sex, among other potential factors.

Our findings are largely in agreement with the limited existing research that has directly examined the relationships between FFM and USG. Hamouti et al. [9] sampled two groups of male athletes, rugby players and endurance runners, and found significantly greater specific gravities, osmolalities, urea concentrations, and uric acid concentrations in urine provided by rugby players. Although not statistically significant, numerically greater urine creatinine concentrations were also found in rugby players compared to endurance runners [9]. In the same study, significant moderate positive correlations were observed between estimated muscle mass and protein metabolites (uric acid + urea + creatinine) (*r* = 0.47) in the urine, while significant, strong positive correlations were observed between USG and these same protein metabolites within urine (*r* = 0.92).

Ehlert and Wilson [8] did not sample athletes specifically but did still find a positive association between estimated FFM and urine osmolality in American adults. Specifically, Americans in the highest tertile of FFM had 2.5 times the odds of elevated urine osmolality as Americans in the lowest tertile of FFM. Further, the authors found that estimated FFM explained 18% of the variance in urine creatinine. A subsequent study by Wilson [10] found that Americans with the most FFM (quintile 5) had USG values that were 0.003–0.004 points higher than those with the least amount of FFM (quintile 1). While Wilson [10] did not report the size of correlation between FFM and USG, *R*^2^ values from regression were reported; among men, the *R*^2^ was 0.026 while it was 0.06 in women. These *R*^2^ values would equate to correlation coefficients of approximately 0.16 for men and 0.25 for women, respectively, which are smaller than the effects observed in the present review. The smaller effect sizes observed in Wilson [10], as compared to the present analysis, could be due to the fact that an estimated FFM value (derived from age, height, mass, waist circumference, and race) was used in that study, while the present review included studies which quantified FFM with more valid methods such as densitometry, dual-energy X-ray absorptiometry, and bioelectrical impedance analysis.

Findings from several studies suggest that USG is impacted by a number of factors, including body composition, body size, and urine solute differences [9, 12]. This study confirms that USG seemingly depends somewhat on a person's FFM amount, and the presence of the muscle metabolite creatinine in the urine is likely partially responsible [10]. Notably, other studies have indicated that additional protein metabolites, urea and uric acid, can increase USG [9]. As byproducts of skeletal muscle degradation, these protein metabolites, specifically urea, may be excreted in greater amounts for those with large amounts of FFM or those consuming more daily protein [13].

The differences in correlation sizes between men and women observed in this study may be reflections of the presence of differences in amounts of urinary creatinine. Men tend to have greater levels of serum creatinine than women with similar urinary excretion rates [14], which may be partly responsible for the weaker relationship between USG and FFM among men. Additionally, women have exhibited lower baseline values for USG compared to men [12], and this, combined with lower levels of serum creatinine, could mean that USG is more greatly affected in women with greater FFM levels.

This study is not free from limitations. First, there were fewer observations for women in general despite inclusion of over 150 original studies. Furthermore, body composition and its variables (i.e., FFM and fat mass) were derived from a variety of methodologies (e.g., skinfolds, bioelectrical impedance, and dual energy X-ray absorptiometry), which result in different FFM and body fat estimates. Additionally, some studies provided USG from a single point in time or sample, while others may have provided several estimates. Depending on the nature of the data and its collection, some USG values were averaged together, while single values were chosen in other cases. Time of day was not consistent between studies either, and lack of any standardization or consistency in time of urine collection may have had an effect given that USG tends to be elevated first thing in the morning [3]. Lastly, the specific approaches (refractometers, benchtop analyzers, and reagent strips) used to quantify USG varied between included studies, which introduces an additional source of measurement error into the analysis.

## 5. Conclusions

This analysis adds to prior evidence that USG is modestly influenced by FFM. These results may be most valuable in sport, where USG is used as a cheap, convenient, and practical field assessment tool to measure hydration status. However, if USG continues to be utilized in these settings, more research is needed to determine the underlying reasons why USG tends to rise with greater FFM levels and if development of FFM-specific USG thresholds may be more appropriate for determining hydration status.

## Figures and Tables

**Table 1:** Number of estimates available for each variable, along with descriptive data by sex.

Men		Women	
	Median (25–75^th^ percentile)		Median (25–75^th^ percentile)
USG (*n* = 171)	1.020 (1.016–1.022)	USG (*n* = 59)	1.017 (1.014–1.020)
Age, y (*n* = 149)	23.8 (20.8–31.0)	Age, *y* (*n* = 48)	22.2 (20.3–28.9)
Mass, kg (*n* = 166)	78.3 (74.7–83.9)	Mass, kg (*n* = 56)	64.0 (61.7–67.2)
Height, m (*n* = 147)	1.79 (1.77–1.82)	Height, m (*n* = 50)	1.67 (1.65–1.70)
Body fat, % (*n* = 93)	13.6 (11.3–15.4)	Body fat, % (*n* = 22)	22.7 (21.4–28.5)
Fat mass, kg (*n* = 91)	9.8 (8.5–12.7)	Fat mass, kg (*n* = 22)	14.0 (13.1–18.3)
BMI, kg/m^2^ (*n* = 152)	24.2 (23.3–25.9)	BMI, kg/m^2^ (*n* = 55)	22.8 (21.9–24.2)
FFM, kg (*n* = 91)	66.7 (63.9–69.7)	FFM, kg (*n* = 22)	46.9 (45.4–49.4)

Abbreviations: BMI, body mass index; FFM, fat-free mass; USG, urine specific gravity.

**Table 2:** Correlations between USG and variables of interest.

	**Age (n = 149)**	**Mass (n = 166)**	**Height (n = 147)**	**% fat (n = 93)**	**FM (n = 91)**	**BMI (n = 152)**	**FFM (n = 91)**

Men USG	−0.34 (< 0.001)	0.24 (0.002)	0.11 (0.178)	−0.08 (0.461)	0.03 (0.766)	0.28 (< 0.001)	0.36 (< 0.001)

	**Age (n = 48)**	**Mass (n = 56)**	**Height (n = 50)**	**% fat (n = 22)**	**FM (n = 22)**	**BMI (n = 55)**	**FFM (n = 22)**

Women USG	−0.57 (< 0.001)	0.03 (0.81)	0.02 (0.885)	−0.31 (0.164)	−0.15 (0.521)	0.04 (0.753)	0.57 (0.006)

*Note:* Spearman's *ρ* is presented along with the *p* value in parentheses.

Abbreviations: BMI, body mass index; FFM, fat-free mass; FM, fat mass; USG, urine specific gravity.

## Data Availability

The data that support the findings of this study are available in the supporting information of this article.

## Supporting Information

Supporting Information 1 contains information extracted from identified studies (type of athlete/exerciser, sample size, and sex distribution, as well as mean values for age, height, body mass, BMI, body fat percentage, FFM, body fat mass, and USG). Other contextual notes about the extracted data, such as the methods/devices used to assess USG and body composition, as well as the specific USG values used in the analysis are also included in Supporting Information 1.
